# Supplementary material for: On metrics for subpopulation detection in single-cell and spatial omics data
Source: Nucleic Acids Res. 2025 Sep 27;53(18):gkaf921. doi: 10.1093/nar/gkaf921 (PMC12476229; doi:10.1093/nar/gkaf921)
Supplement: gkaf921_Supplemental_File [file gkaf921_supplemental_file.pdf]

Supplementary Figures

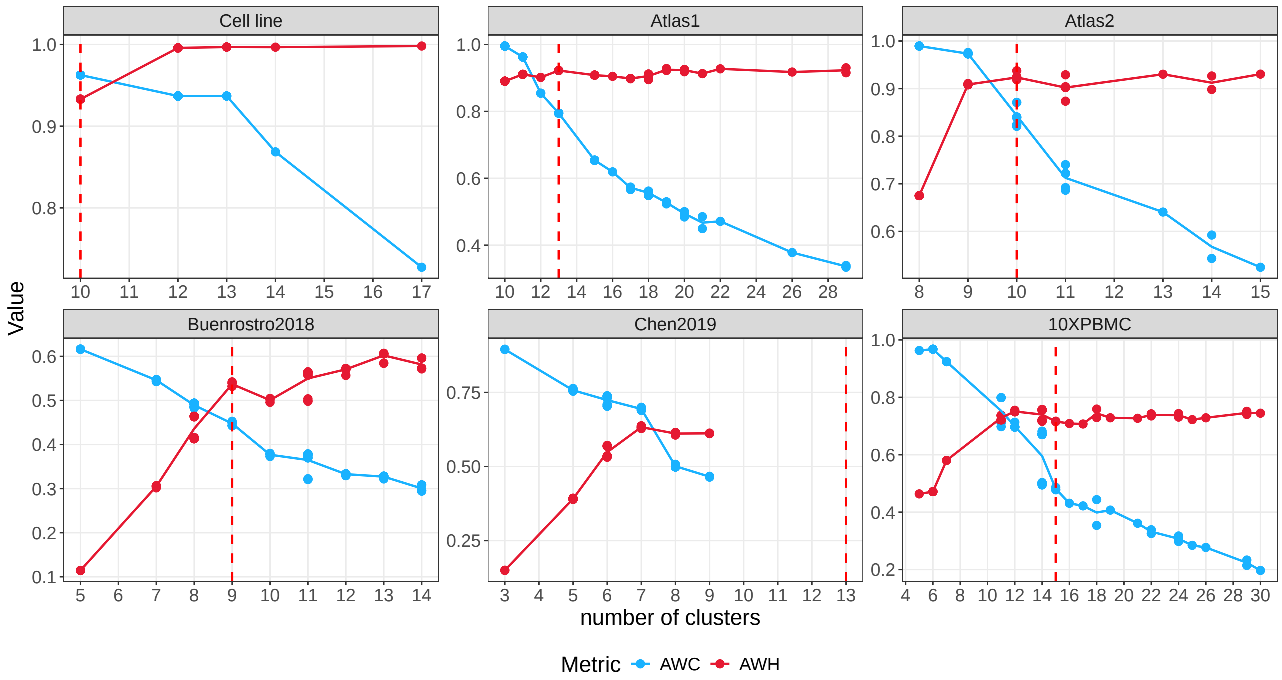

Figure S1. The trade-off between AWH and AWC as the number of clusters varies. Here six single-cell ATAC-seq datasets are used for benchmarking clustering. The red dash line is the number of classes of the ground truth. Each dot is a clustering solution. Different numbers of ATAC clusters are achieved by applying a range of resolutions during Leiden clustering. See [1] for more details.

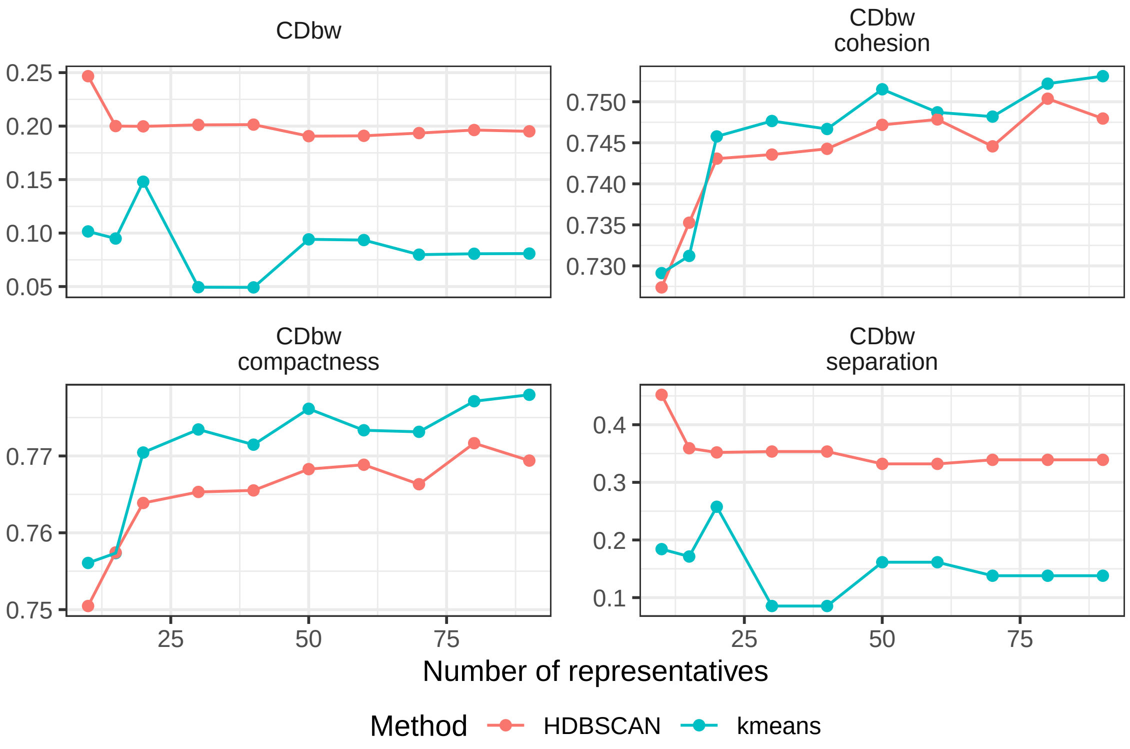

Figure S2. How the number of representative points influence CDbw metrics. In relation to Figure **??** c-d, we calculate the CDbw metrics using a variety of representative numbers. Similar to Figure **??** d where 10 representatives are used, the CDbw and CDbw separation metrics indicate that HDBSCAN produces a better clustering than kmeans for this toy example. However, the CDbw cohesion and compactness metrics fail to capture this distinction and rank them oppositely.

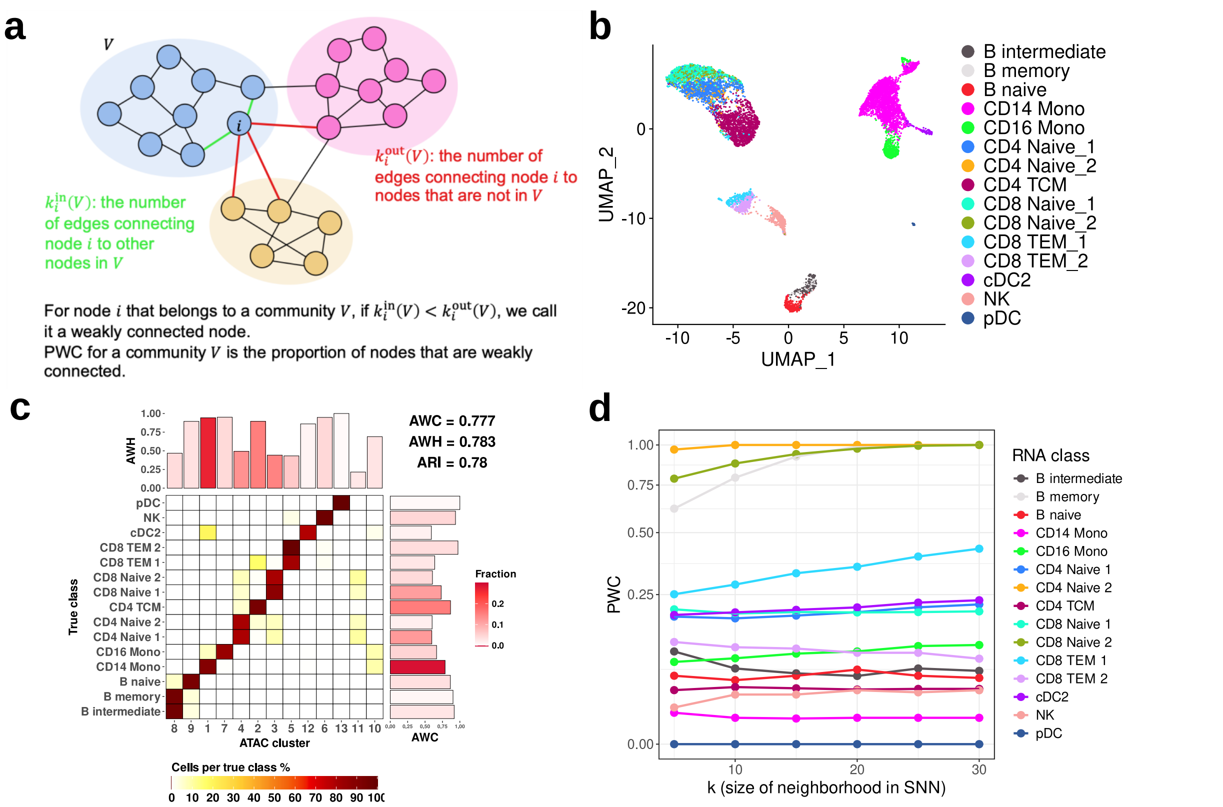

Figure S3. Illustration of the utility of PWC in single-cell datasets. a Schemetic overview of how PWC is calculated. **b** The UMAP of this dataset. **c** An example clustering results showing the classes that are failed to be seperated. **d** The value of PWC across a range of k when building the SNN graph.

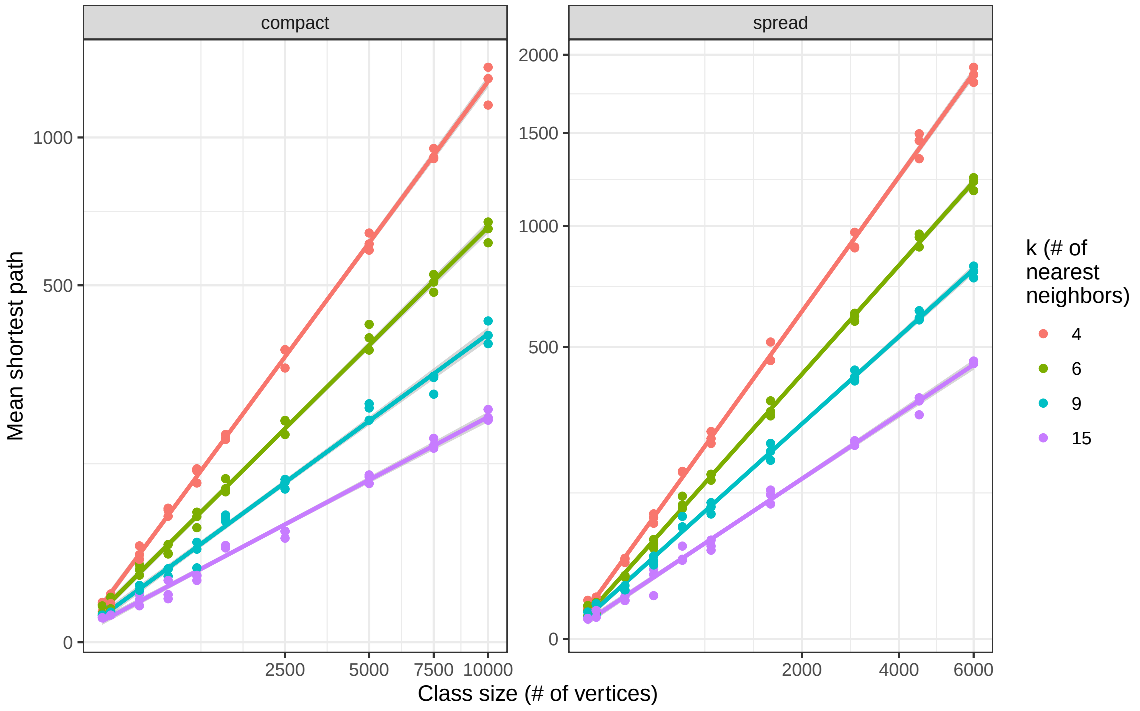

Figure S4. The mean shortest path (in steps) is linearly proportional to the number of vertices. Shown are simulated two-class graphs containing a more compact and more spread class, simulated with the same parameters except for a size factors. Each size is simulated with 3 random seeds.

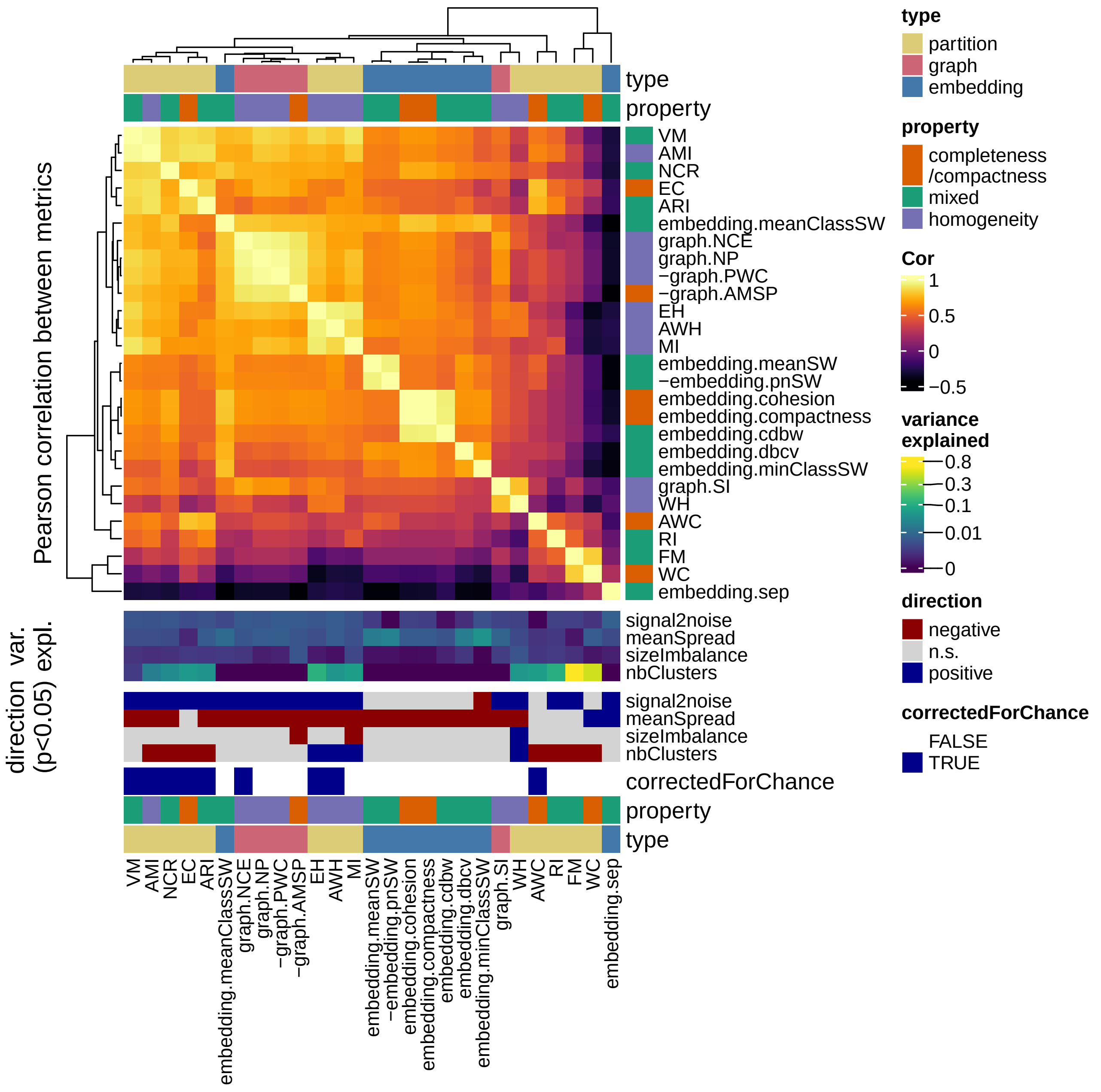

Figure S5. Comparison of metrics across data representations. As explained in Supplementary Information 1, 2240 simulated datasets of 3 or 4 classes with varying abundance, variability, and inter-class distances are used. The main heatmap shows the Pearson's correlation coefficients between metrics. The bottom heatmaps are the percentage of variance explained by each simulation factor using a linear regression model, the effect directions, and the metric categories, respectively.

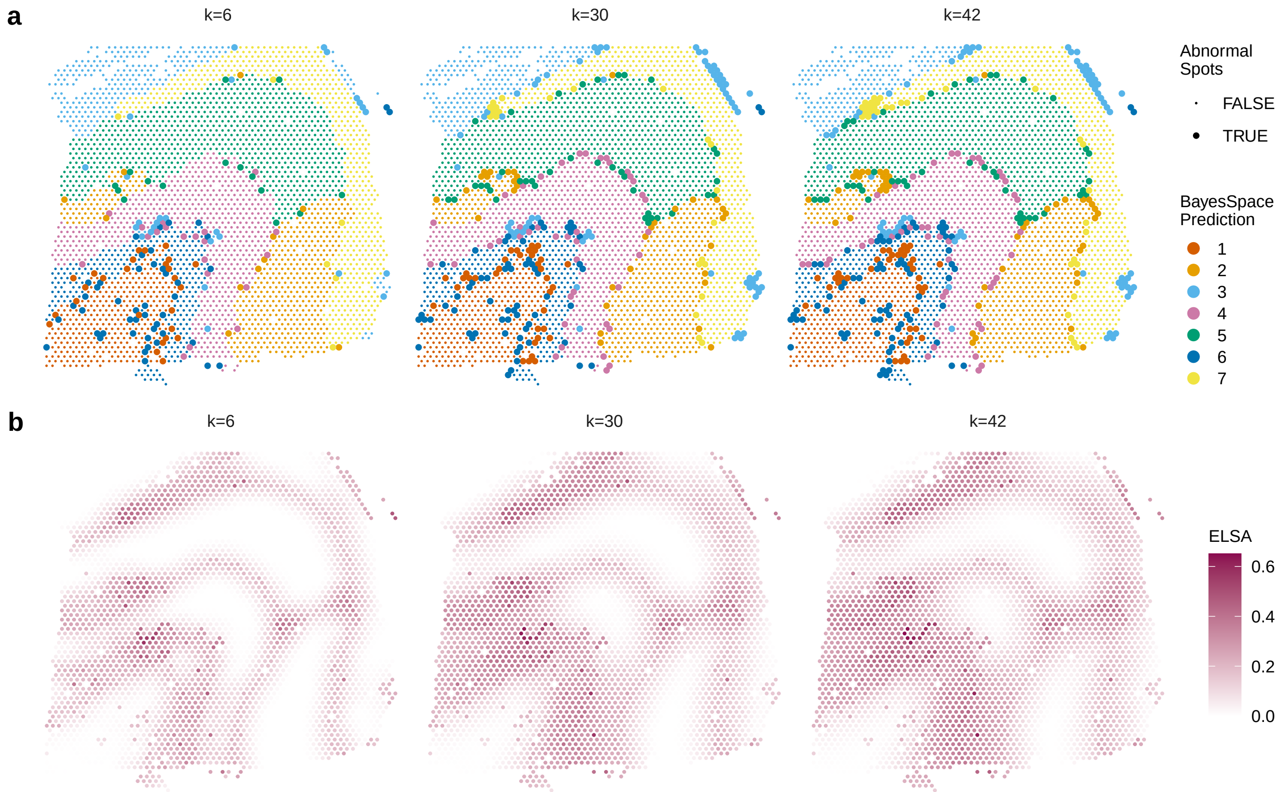

Figure S6. Element-level internal metrics as the size of neighborhood changes. This is related to Figure **??**. k is the size of the neighborhood used to calculate the element-level metric values. Score in **a** indicates whether a spot is classified as abnormal or not; Score in **b** is the ELSA values.

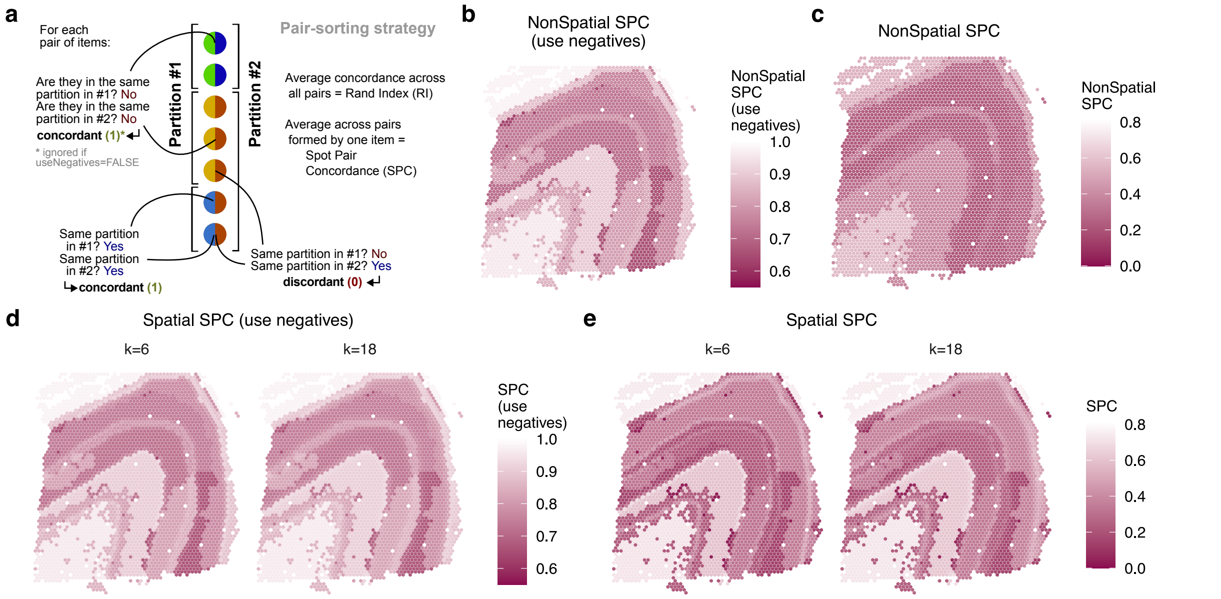

Figure S7. Variants of the Spot-wise Concordance (SPC) score. a Illustration of the pair-counting strategy. **b-e** show the values of SPC variants for BayesSpace's prediction as in Figure **??**. **b-c**: SPC not accounting for spatial relationships, and using (**b**) or not using (**c**) negative pairs. **d-e**: Spatially-aware SPC using (**d**) or not using (**e**) negative pairs, and across different neighborhood sizes (k).

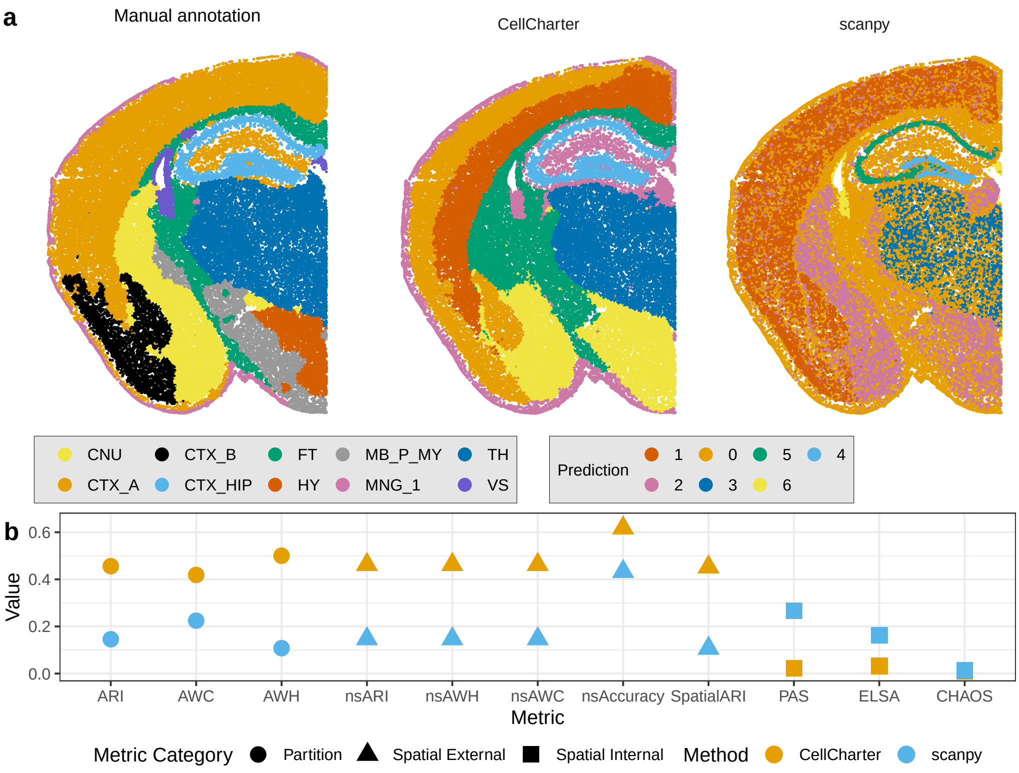

Figure S8. The same metrics are applicable to imaging-based spatial data. To illustrate, the STARmap PLUS mouse brain dataset (slice 11, Shi et al. [2]) is used. In **a**, the first panel shows manual annotations, while the second and third panels show domain predictions from two clustering methods: CellCharter, which is spatially aware, and scanpy, which is not. In **b**, representative partition-based metrics, as well as spatial external and internal metrics are calculated.

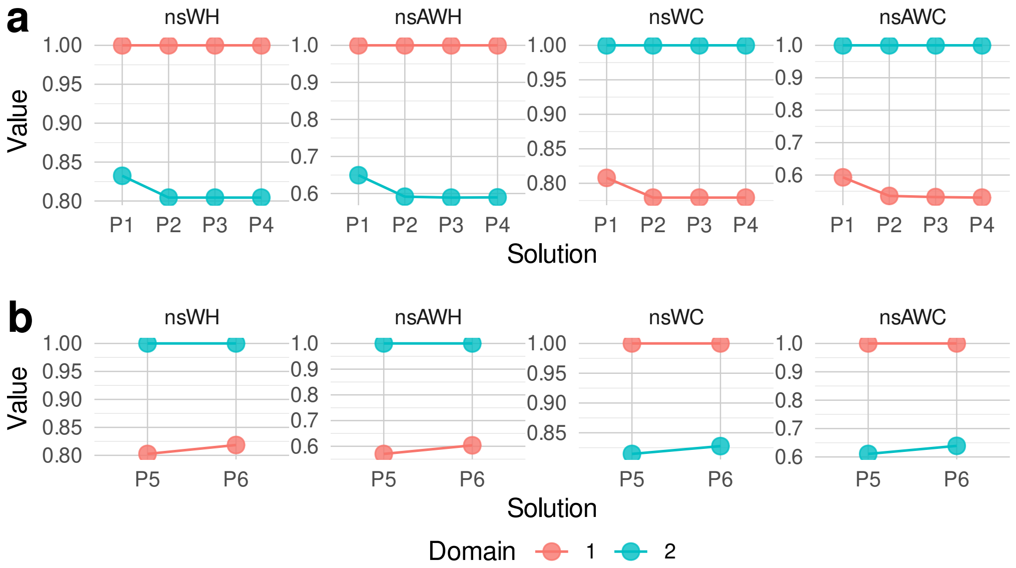

Figure S9. The values of domain-level partition metrics. Related to Figure **??**. The cluster-specific (fuzzy-hard version of) spatial WH and AWH, as well as class-specific (fuzzy-hard version of) spatial WC and AWC are calculated for P1-P3 in **a**, and for P4-P5 in **b**, respectively.

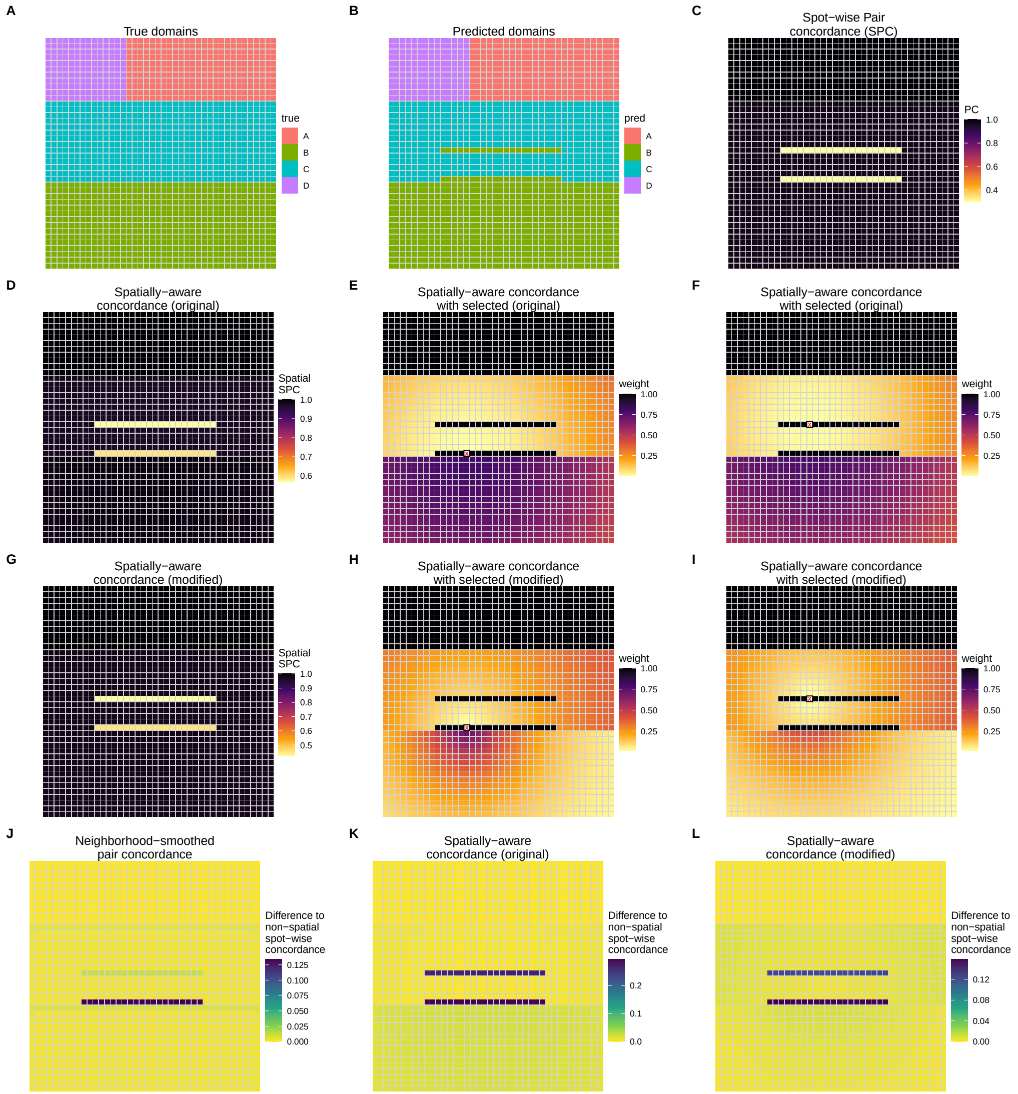

Figure S10. A toy spatial dataset to illustrate the original and adapted distance functions. **a** True domains. **b** Predicted domains. **c** Non-spatial spot-level mean pair concordance (SPC), showing the spots with disagreement between predicted and true domains. **d** Spatially-aware spot concordance, computed using the original distance-weighted functions from Yan et al. [3]. The spot-level differences are very mild, and no difference between the two misclassified stripe can be observed at least by eye. **e-f** Illustration of the distance decay for two different highlighted spots. The colors indicate the (distance-weighed) concordance between each spot and the highlighted spot (marked with an X). While the concordance patterns match the intuition of tolerating the wrong grouping of nearby spots, we believe that this understanding of ‘nearby’ is much to loose, ending up making errors across most of the field of view tolerated. **g-i** Same as **d-f**, but using the adjusted distance functions, which we deem more reasonable. **j-l** The difference between the three spatially-aware spot-wise concordance and the non-spatial one. The neighborhood-smoothed concordance shows good edge tolerance, while the original distance-weighted ARI from Yan et al. [3] does not: the two stripes show a similar increase in concordance from the spatial weighing. In contrast, our modified spatial ARI recovers the difference between the two stripes.

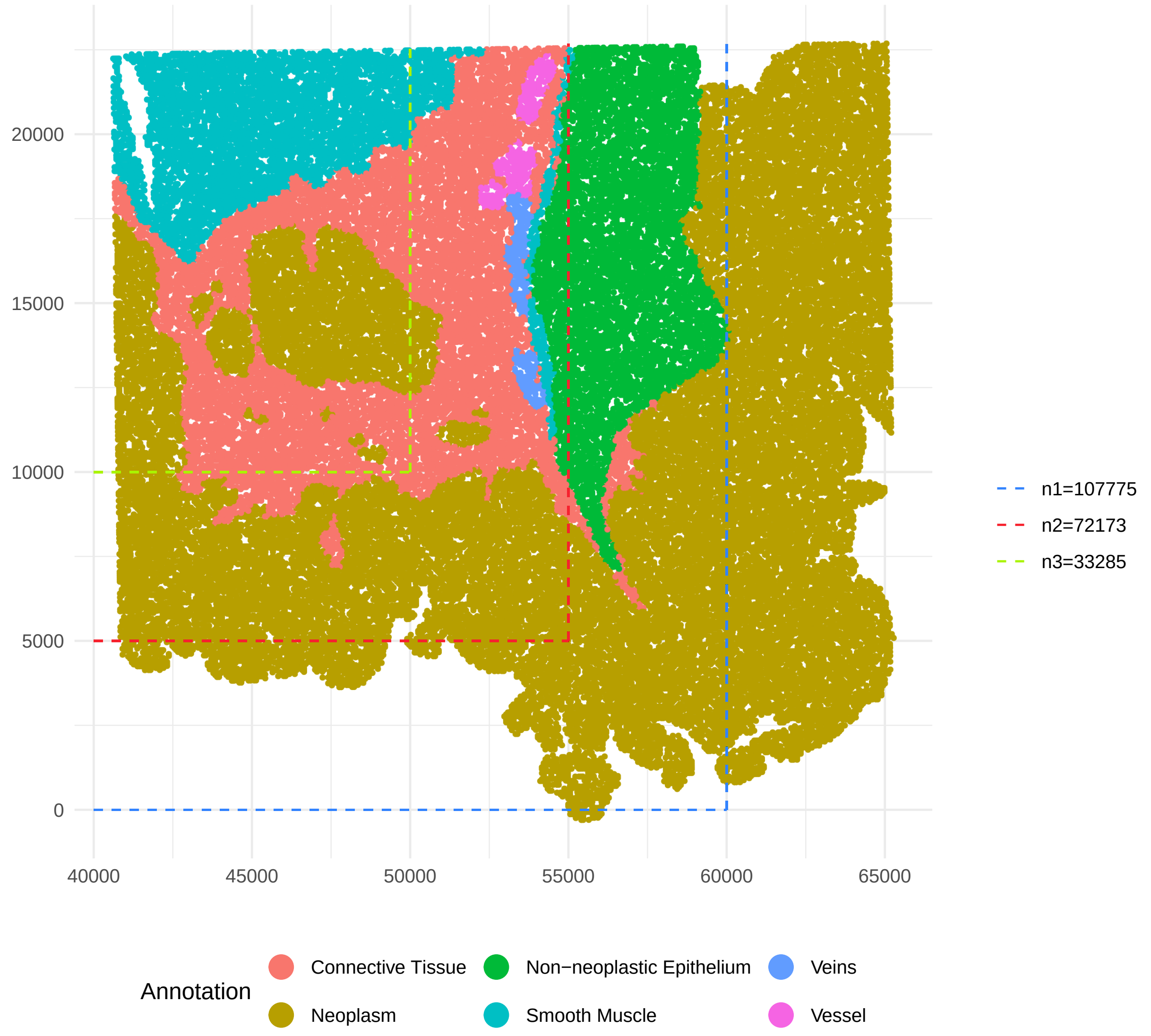

Figure S11. Datasets used for scalability monitoring. The Visium HD colon cancer dataset from [4] was used to monitor CPU time and memory usage in Figure S12. The full slide contains 136,870 cells. To assess scalability, we performed three rounds of subsampling by selecting subregions of the slide, as indicated by the dashed lines, resulting in datasets with 107,775, 72,173, and 33,285 cells, respectively. Each selected metric was then calculated across the three subsampled datasets and the full dataset, repeated five times for each. Cells are colored by the manual annotation from the original paper.

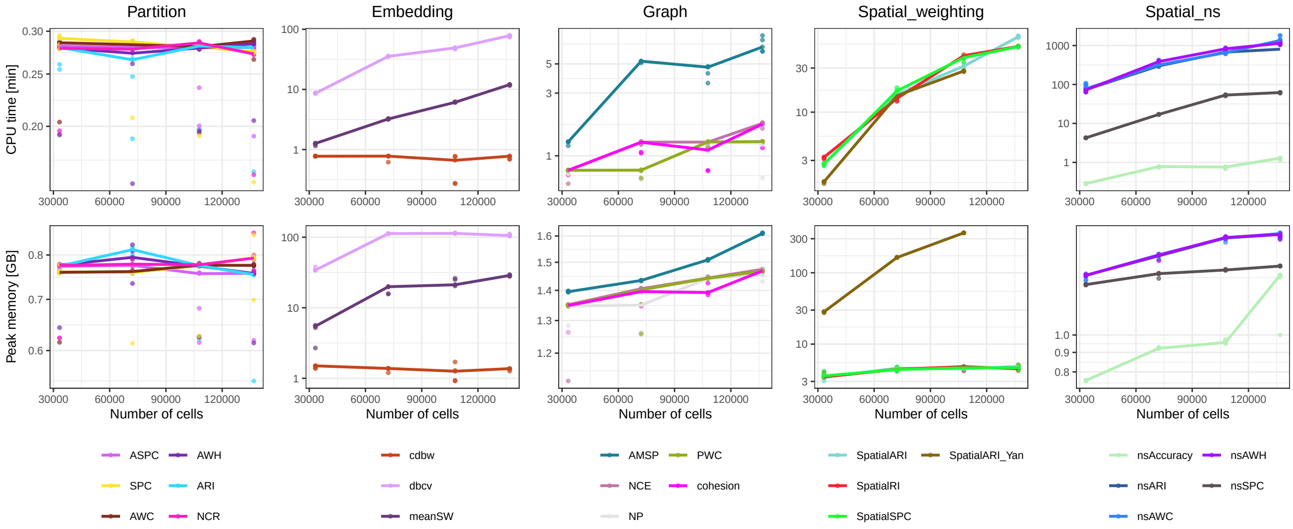

Figure S12. The CPU time and peak memory usage of each selected metric across datasets of different sizes. The Visium HD colon cancer dataset [4] and its subregions (see Figure S11) were used to monitor the runtime and memory usage. Each selected metric was computed on all four dataset sizes, with five repeated runs per size. The individual runs are shown as dots, and the median values across the five runs were used to generate the line plots. The y-axes are presented on a logarithmic scale. The selected metrics are grouped into five categories, each shown in one panel: partition-based, embedding-based, and graph-based metrics, as well as spatial metrics based on distance-weighting (Spatial_weighting), and those based on neighborhood smoothing (Spatial_ns).

Bibliography

1. Siyuan Luo, Pierre-Luc Germain, Mark D Robinson, and Ferdinand von Meyenn. Benchmarking computational methods for single-cell chromatin data analysis. *Genome Biology*, 25(1):1–30, 2024.
2. Hailing Shi, Yichun He, Yiming Zhou, Jiahao Huang, Kamal Maher, Brandon Wang, Zefang Tang, Shuchen Luo, Peng Tan, Morgan Wu, et al. Spatial atlas of the mouse central nervous system at molecular resolution. *Nature*, 622(7983):552–561, 2023.
3. Yinqiao Yan, Xiangnan Feng, and Xiangyu Luo. Spatially Aware Adjusted Rand Index for Evaluating Spatial Transcriptomics Clustering. *bioRxiv*, page 2025.03.25.645156, March 2025. doi: 10.1101/2025.03.25.645156. URL https://www.biorxiv.org/content/10.1101/2025.03.25.645156v1. Publisher: Cold Spring Harbor Laboratory Section: New Results.
4. Michelli Faria de Oliveira, Juan Pablo Romero, Meii Chung, Stephen R Williams, Andrew D Gottscho, Anushka Gupta, Susan E Pilipauskas, Seayar Mohabbat, Nandhini Raman, David J Sukovich, et al. High-definition spatial transcriptomic profiling of immune cell populations in colorectal cancer. *Nature Genetics*, pages 1–12, 2025.
